# Quantitative measurement of resistance force and subsequent attenuation during passive isokinetic extension of the wrist in patients with mild to moderate spasticity after stroke

**DOI:** 10.1186/s12984-022-01087-3

**Published:** 2022-10-13

**Authors:** Kentaro Kawamura, Seiji Etoh, Tomokazu Noma, Ryota Hayashi, Yuiko Jonoshita, Keisuke Natsume, Seiichi Niidome, Yong Yu, Megumi Shimodozono

**Affiliations:** 1 Department of Rehabilitation and Physical Medicine, Graduate School of Medical and Dental Sciences, Kagoshima University, 8-35-1, Sakuragaoka, Kagoshima City, Kagoshima 890-8520 Japan; 2 Department of Rehabilitation, Faculty of Health Science, Nihon Fukushi University, Aichi, Japan; 3 Department of Mechanical Systems Engineering, Okayama University of Science, Okayama, Japan; 4 Department of Rehabilitation, Kagoshima University Hospital, Kagoshima, Japan; 5 Department of Mechanical Engineering, Graduate School of Science and Engineering, Kagoshima University, Kagoshima, Japan

**Keywords:** Muscle spasticity, Stroke, Wrist, Finger, Rehabilitation, Biomechanics, Resistance force, Force attenuation, Objective assessment

## Abstract

**Background:**

Spasticity is evaluated by measuring the increased resistance to passive movement, primarily by manual methods. Few options are available to measure spasticity in the wrist more objectively. Furthermore, no studies have investigated the force attenuation following increased resistance. The aim of this study was to conduct a safe quantitative evaluation of wrist passive extension stiffness in stroke survivors with mild to moderate spastic paresis using a custom motor-controlled device. Furthermore, we wanted to clarify whether the changes in the measured values could quantitatively reflect the spastic state of the flexor muscles involved in the wrist stiffness of the patients.

**Materials and methods:**

Resistance forces were measured in 17 patients during repetitive passive extension of the wrist at velocities of 30, 60, and 90 deg/s. The Modified Ashworth Scale (MAS) in the wrist and finger flexors was also assessed by two skilled therapists and their scores were averaged (i.e., average MAS) for analysis. Of the fluctuation of resistance, we focused on the damping just after the peak forces and used these for our analysis. A repeated measures analysis of variance was conducted to assess velocity-dependence. Correlations between MAS and damping parameters were analyzed using Spearman’s rank correlation.

**Results:**

The damping force and normalized value calculated from damping part showed significant velocity-dependent increases.

There were significant correlations (*ρ* = 0.53–0.56) between average MAS for wrist and the normalized value of the damping part at 90 deg/s. The correlations became stronger at 60 deg/s and 90 deg/s when the MAS for finger flexors was added to that for wrist flexors (*ρ* = 0.65–0.68).

**Conclusions:**

This custom-made isokinetic device could quantitatively evaluate spastic changes in the wrist and finger flexors simultaneously by focusing on the damping part, which may reflect the decrease in resistance we perceive when manually assessing wrist spasticity using MAS.

*Trial registration* UMIN Clinical Trial Registry, as UMIN000030672, on July 4, 2018

## Background

Spasticity is a common complication of various neurological diseases and lesions in the central nervous system [1–3]. It occurs in 17–46% of stroke patients [4] (17%/1 year [5], 42%/6 months [6], 46%/1 year [7]) within 12 months after onset. Spasticity is primarily observed in the elbow (79% of patients), wrist (66%), and ankle (66%) [8].

Spastic symptoms can induce pain, contractures, abnormal posture, decreased range of joint motion, tendon retraction, and muscle weakness in patients, which may also impair the patient’s quality of life and limit the potential success of rehabilitation [9–11]. Accordingly, spasticity management is an essential concept in neurological rehabilitation, and spasticity needs to be measured accurately.

The Modified Ashworth Scale (MAS) measures the level of resistance to passive movement. It is most widely used for evaluating spasticity in a clinical setting and has been investigated in many studies [12], in which the assessor subjectively graded the resistance to manual passive stretch. The MAS grades spasticity as follows: 0 = no increase in muscle tone; 1 = slight increase in muscle tone, manifested by a catch and release or by minimal resistance at the end of the range of motion when the affected part is moved in flexion or extension; 1 +  = slight increase in muscle tone, manifested by a catch followed by minimal resistance throughout the remainder (less than half) of the range of motion (ROM); 2 = more marked increase in muscle tone through most of the ROM, but the affected part is easily moved; 3 = considerable increase in muscle tone, passive movement is difficult; 4 = affected part is rigid in flexion or extension [13]. The MAS is easy to use in clinical practice because it takes little time and no equipment is needed to assess muscle tone [14, 15].

While there have been reports that the MAS shows poor reliability between raters [16–18], several other studies have reported good intra- and inter-rater reliability, mostly for the upper extremities [13, 19, 20], and better reliability for evaluations of the wrist flexor compared to those in the proximal elbow flexor or shoulder adductor [21].

Lance [22] defined spasticity as “a motor disorder characterized by a velocity-dependent increase in tonic stretch reflexes with exaggerated tendon jerks, resulting from hyperexcitability of the stretch reflex” that causes increased muscle tone and, subsequently, increased stiffness to restrict movement. On the other hand, when measuring spasticity in daily clinical practice, especially in patients with mild to moderate spasticity, we often feel not only an increase in muscle tone during passive movement, but also a subsequent sudden decrease in resistance force. This is known as the clasp-knife phenomenon [23]. In addition, in the definition of MAS, "catch and release" and "catch followed by minimum resistance" are mentioned. This sudden decrease in resistance after an increase in resistance during passive extension of the target muscle is a major characteristic of spasticity in patients with mild to moderate spasticity and is a noteworthy change.

To measure and evaluate spasticity more objectively, various quantitative approaches have been tested using different methodologies [24]. Several motor-driven or manually driven devices have been used in patients with stroke, and passive resistance forces have been measured in the ankle [25–29], elbow, [30–33] wrist [34–37], and finger [38] joints. Several studies have shown a significant correlation between the MAS and increased resistance to passive movement in the wrist joint [35, 36]. However, no studies have investigated the correlation between the MAS and force attenuation following increased resistance in the wrist joint when measuring spasticity in patients with stroke.

Since most patients with spasticity after stroke in previous studies showed consistent stretch reflexes at less than 100 deg/s in wrist flexor muscles [34] and at 30–60 deg/s in elbow or ankle joints [26, 39, 40], we thought it would be possible to measure spasticity with the instrument at speeds slower than those typically used. Furthermore, we wanted to avoid the appearance of pain after repetitive measurements.

We have developed a device that can measure passive resistance at low angular velocities and reduces excessive resistance to the wrist joint due to extension. We also focused on and analyzed the characteristic damping force observed after the maximum resistance with extension, which could reflect the decrease in resistance we sense while measuring MAS.

We considered that a safe and easy-to-use device that can evaluate such resistance may be useful in introducing the objective measurement of spasticity into daily clinical practice. The purpose of this study was to investigate whether a custom-made motor-controlled device could be used to safely measure extension stiffness in a relatively mild spastic wrist joint in a patient with a post-stroke hemiparetic upper limb. Furthermore, we wanted to clarify whether the subsequent damping changes after the peak resistance could quantitatively reflect the spastic state of the wrist flexors and extrinsic finger flexors involved in wrist stiffness by examining its correlations to the MAS.

## Materials and methods

### Study design

This was a cross-sectional study with a single test session. In subjects with post-stroke hemiparesis (N = 17), the spasticity of the wrist flexor and finger flexor muscles was assessed by two raters using MAS as a clinical assessment. On the other hand, resistance force during passive wrist extension was measured using a device that consisted of a custom-designed hand and forearm plate with a force sensor and a servo-controlled DC torque motor for objective assessment. The resistance force from wrist flexion (20° palmar flexion) to extension (90% of extreme dorsiflexion) was measured for 11 cycles at velocities of 30 deg/s, 60 deg/s, and 90 deg/s. The obtained 10 measures, excluding the first, were used in the analysis. Intra-rater reliabilities, velocity dependence, correlation with the average MAS, and differences depending on the severity of spasticity (subjects were divided into two groups of mild and moderate spasticity depending on the average MAS score) were investigated.

### Outcome measures

The resistance force in the change from wrist flexion to extension was defined as the maximum resistance force (“**maximum RF”**). After maximum wrist extension, the peak resistance force and a subsequent decrease in resistance force were observed. We used the term “damping” to describe this remarkable decrease. We defined the force-gap between the peak resistance force and the subsequent most strongly decreased resistance force as the “**damping force**”. We calculated the impulses from the timing of the peak to that of the most attenuated, damped resistance. We defined the area of only the changed part from the peak force as the “**pure damping impulse”**, and that of the entire damping area as the “**total damping impulse”**. We defined the ratio of the damping force to the maximum RF as the “**damping force ratio”** and the ratio of the pure damping impulse to the total damping impulse as the “**damping impulse ratio”**.

MAS scores obtained by the two therapists were averaged for wrist and finger flexors to give an “**average MAS**”. The average MAS scores of wrist and finger flexors combined were further divided and an average value per joint was calculated.

### Participants

The patients were recruited from January 2018 to March 2019. These patients were inpatients or outpatients with stroke from Kagoshima University Hospital and Kagoshima University Hospital Kirishima Rehabilitation Center, Japan. The inclusion criteria were as follows: (1) age between 16 and 80 years, (2) the presence of hemiparesis in an upper limb with stroke, and (3) wrist and finger joint spasticity in the range of MAS 0–3 (out of 0–4; 0, 1, 1 +, 2, 3, and 4) in the hemiparetic upper limb. Participants were excluded if they had pain in the upper limb or could not understand the study or simple commands.

All of the subjects gave their written and oral informed consent to the experimental procedures and the study. The study was approved by the ethics committee of Kagoshima University Hospital (Study Number: 170201) and was performed in accordance with the Declaration of Helsinki.

### Apparatus

**Fig. 1:**
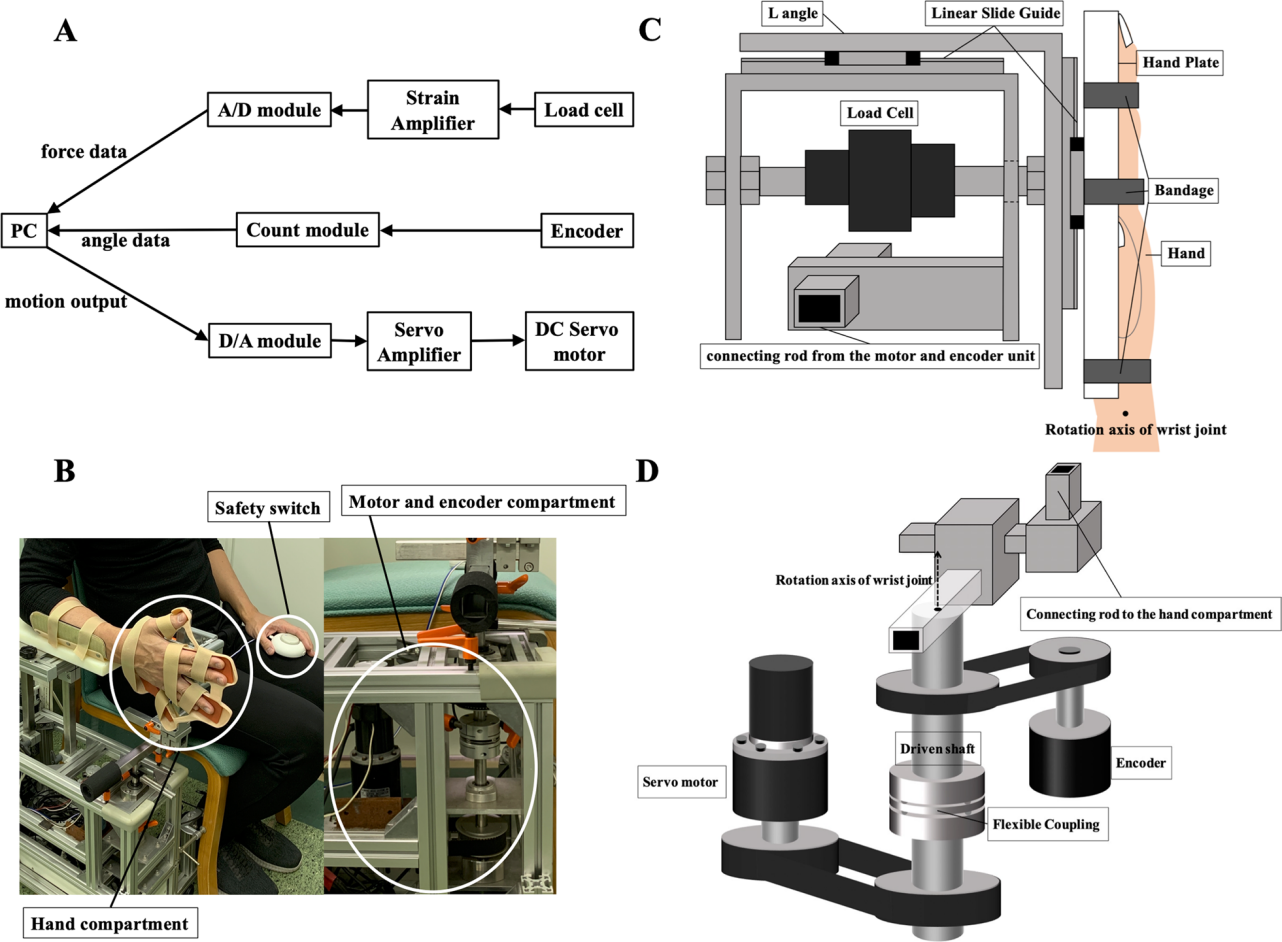
Motor-controlled isokinetic custom apparatus to measure the extension stiffness in wrist joint. **A** Configuration of the experimental setup. **B** Appearance of the device. Left side: Overall picture of the equipment and positioning of the forearm and hand compartment. The hands were fixed with all fingers extended to reflect not only the spasticity of wrist flexors but also that of the extrinsic finger flexors. Right side: Lateral view of the motor and encoder compartment. **C** Top view of the hand compartment: The force sensor with a sliding system and hand plate. **D** Detailed view of the motor and encoder compartment. The axis of rotation (dotted arrow) was aligned with the axis of the wrist joint (solid circle in **C**)

The device consisted of a custom-designed hand and forearm plate with a force sensor; Tension/Compression load cell (LUR-A-SA1: Kyowa Electronic Instruments Co. Ltd, Tokyo, Japan) and a servo-controlled DC torque motor (RH-14D-3002: Harmonic Drive Systems Inc., Tokyo, Japan). The outputs from the transducers were amplified and displayed on a laptop PC. The position and force data were recorded with a sampling rate of 66 Hz. The experimental setup and the equipment are shown in Fig. 1A and B. The load cell was attached to a bracket connected to the rod from the motor. It was mounted to be perpendicular to the hand orthosis attached to the L-shaped angle material. The other end of the load cell was fixed to the L-shaped angle material through a hole in the bracket. The slide guide on the upper part of the load cell moves horizontally due to the tensile/compressive force from the hand, and the force is transmitted to the load cell to measure the resistance of the wrist joint. By attaching a slide guide to the side of the hand plate (Fig. 1C), it was expected that unnaturally excessive forces would not be applied to the wrist joint. Figure 1D shows an enlarged detailed view of the motor and encoder compartment.

### Equipment for safety measures

Excessive force loading on the wrist joint may cause persistent pain, and we took special care to prevent such loading in this study. The device was designed so that rotation of the wrist joint could be stopped by a force of 90 N or more. Furthermore, when the resistant force measured by the load cell exceeded 50 N, the control software was programmed to automatically stop the motion of the rotating part of the device. Since the normal hand strength range is typically 300–500 N [41], these safety limits of resistant force were designed to allow a nearby examiner to easily stop the device during the examination. The upper limit of this force could be arbitrarily changed to a small value depending on the subject. In addition, a safety switch (Fig. 1B) was provided to the subjects during the examination so that the device could be stopped immediately if they felt any discomfort or pain.

### Experimental procedures

Subjects were comfortably seated upright on a chair and evaluated for muscle spasticity using the MAS prior to each experiment by two therapists who had been well trained and had more than several years of experience in MAS evaluation. To ensure the independence of the evaluation, each evaluator assessed spasticity separately so that they would not know each other's findings. The two therapists assessed the spasticity of wrist flexor and finger flexor muscles using MAS, which is classified into six levels [13, 42]. MAS scores of 0–4 (0, 1, 1 + , 2, 3, and 4) were assigned numerical values of 0–5 (0, 1, 2, 3, 4, and 5, respectively) for data analysis. These MAS scores obtained by the two therapists were averaged for wrist and finger flexors, to give an “average MAS” [43, 44]; minimum 0 to maximum 5.

For force measurements, subjects were comfortably seated beside a servo-controlled DC torque motor. Each subject’s forearm was fixed in an adjustable arm support and the hand with all fingers extended was strapped to the hand plate coupled to a Tension/Compression load cell. The initial posture of the subjects was set so that the shoulder was in the neutral position, the elbow was flexed at 90°, the forearm was in the neutral position, and the hand and forearm were initially positioned at a relative angle of 0°; this was set as the neutral wrist position (Fig. 1B). The rotation axis of the device was aligned to the anatomical axis of the wrist joint by sliding the hand plate and forearm holder. The forearm and fingers were fastened to the device by using Velcro straps, and the examiner checked to ensure that the participants felt no pain or discomfort in the neutral fastened position. The overall experimental procedure consisted of two steps. First, the examiner manually moved the hand plate slowly from the neutral wrist position to the position of extreme dorsiflexion, then moved it to the 20° palmar flexed position. Ninety percent of extreme dorsiflexion (from the neutral wrist position) was set as the maximum range of extension so as not to induce pain. In the procedure, the laptop PC sampled and displayed the angular position of the wrist joint. In the second step, 11 cycles of passive extension were performed for force measurements at three angular velocities in the following order, 30 deg/s, 60 deg/s, and 90 deg/s. A stretch cycle was defined as from 20° flexion (palmar flexion) to maximum extension (dorsiflexion) at the previously mentioned velocity, and then back to 20° flexion at a velocity of 15 deg/s. Measurements at each extension velocity were obtained for 11 cycles of passive extension and flexion with 2-s pauses at the end of each movement. Each cycle (with different angular velocities) was performed with a 1- to 2-min rest period between the cycles.

### Data analysis and statistics

**Fig. 2:**
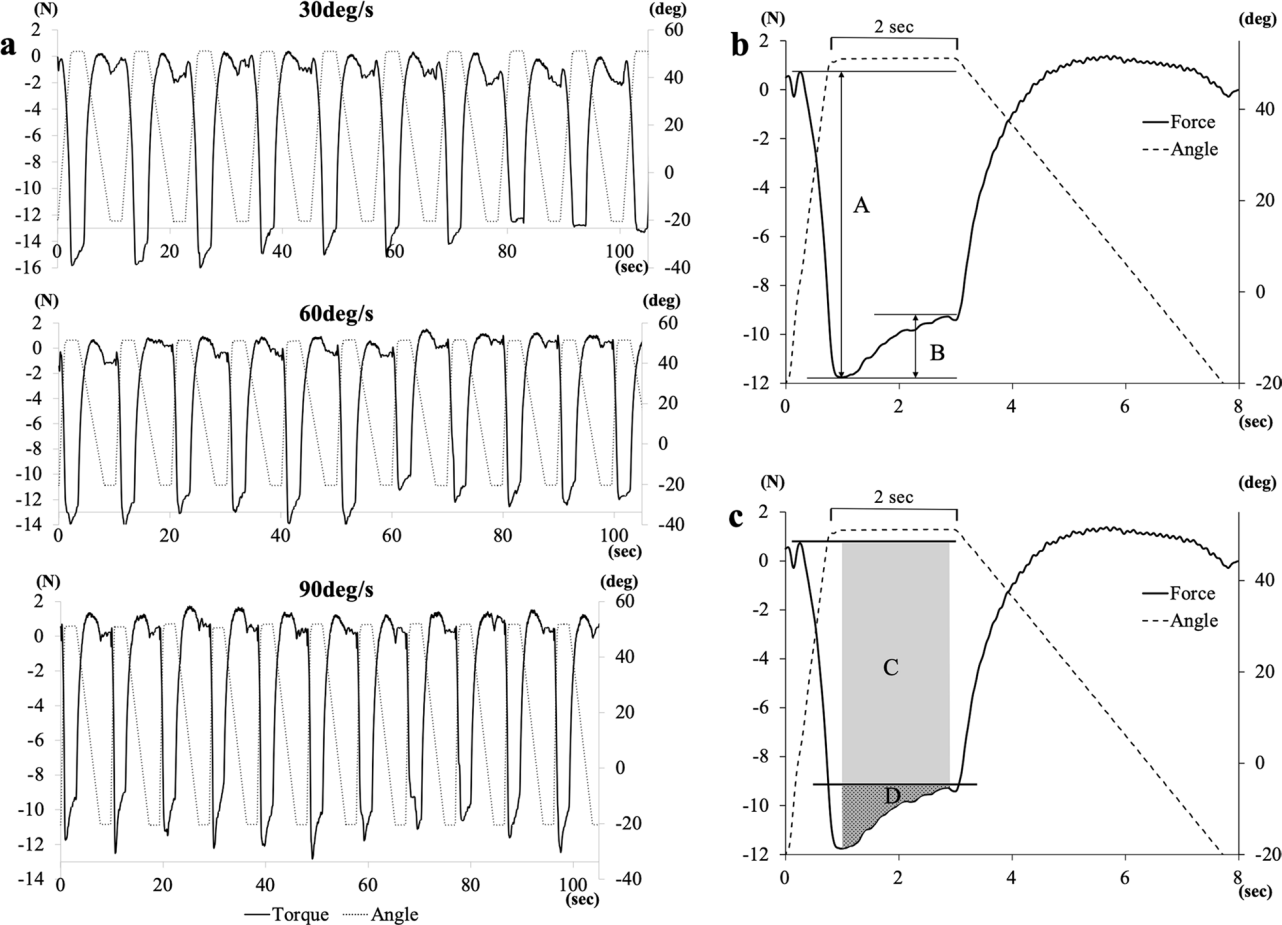
Waveforms obtained by measurement and definitions. **a** Typical example of changes in resistance force and angular displacement in the wrist joint at each of three angular velocities (30, 60, 90 deg/s) with sequential measuring during 11 repetitions. **b** The difference between the peak forces (i.e. between the maximum and minimum forces) immediately after the start of extension was defined as the maximum resistance force (RF) (A). The force attenuation during the subsequent 2-s maintenance of the extended position was defined as the damping force (B). **c** The area from the timing of the peak resistance to the timing of the greatest attenuation of resistance was defined as the pure damping impulse (D), and the entire damping area under the curve (including the pure damping impulse) was considered the total damping impulse (C)

Of the 17 subjects, most (15 cases) had a peak resistance force during wrist extension as a negative value. The other two cases had positive peak resistance forces. We considered that the resistance force transmitted to the load cell changed to the proximal side (minus) or the distal side (plus) of the load cell axis due to the sliding mechanism of the hand fixture. Typical examples of 11 cycles of resistance force–time curves with repetitive angular displacement of wrist extension and flexion at each angular velocity obtained with the device are shown in Fig. 2a. The first recording of each of 11 repetitive measurements was excluded from the analysis to avoid bias from startle reflexes.

An example of an enlarged force–time curve with angle data extracted from repetitive measurements is shown in Fig. 2b and c. For each curve, the resistance force in the change from wrist flexion to extension was defined as the maximum resistance force (maximum RF) (“A” in Fig. 2b). On the other hand, after the peak resistance force (negative peak)was reached just after the maximum angle, a notable decrease in resistance force (in the positive direction) was observed (“B” in Fig. 2b); namely, the force-gap between the peak resistance force and the subsequent most strongly decreased resistance force was defined as the “damping force” (B). Furthermore, the impulses from the timing of the peak resistance to the timing of the most attenuated resistance were calculated. We defined the area of only the changed part from the peak force as the pure damping impulse (“D” in Fig. 2c), and that of the entire damping area under the curve (including the area of the pure damping impulse) as the total damping impulse (“C” in Fig. 2c). The trapezoidal integration rule was used to approximate the definite integral for calculating impulses.

In previous reports (on the ankle joint), some normalization processes were used to highlight differences [26, 27]. In these reports, normalized values were obtained by expressing passive resistance as a percent of the torque values measured at an extremely low velocity (10 deg/s) [27], or of the torque values evoked with supramaximal stimulation of the tibial nerve [26]. With reference to these previous studies, we applied normalization to the obtained values. We defined the ratio of the damping force (B) to the maximum RF (A) as the damping force ratio (B/A) (Fig. 2b) and the ratio of the pure damping impulse (D) to the total damping impulse (C) as the damping impulse ratio (D/C) (Fig. 2c). The absolute value was obtained, and the mean values of repetitions were used for subsequent data analysis.

For assessment of the intraclass correlation coefficient (ICC), Shrout and Fleiss classified the ICC into three types (Cases 1 to 3) [45]. Since tests with the isokinetic device and force measurements were supervised by one rater in this study, the intra-rater reliabilities of 10 repetitive measurements of various parameters were computed with Case 1 (ICC [1, 1] and ICC [1, 10]) for each angular velocity. The reliability calculated with ICC is considered to be excellent for values from 0.75 to 0.90, fair to good for values between 0.40 and 0.75, and poor for values less than 0.40 [46]. To assess the agreement in MAS between the two raters, we used Cohen’s kappa coefficient.

The normality and equality of variance were checked by the Shapiro–Wilk and Levene statistical tests. According to these results, a two-sample t-test (TT) or Wilcoxon-Mann–Whitney test (WMW) was performed to compare the differences between the mild (average MAS ≦ 1) and moderate (average MAS > 1) spastic groups. A repeated measures analysis of variance (ANOVA) was conducted to assess velocity-dependent differences. Friedman’s test followed by the Wilcoxon signed-rank test with Holm’s correction was conducted to evaluate differences for angular speeds of 30 deg/s, 60 deg/s, and 90 deg/s. Correlations between MAS and maximum RF and damping parameters were analyzed using Spearman’s rank correlation. All statistical analyses were performed using **R Commander** software 2.7–0 (R4.0.2; CRAN, freeware). The significance level for all statistical tests was set to 0.05.

## Results

Table 1 shows the characteristics of the patients. Seventeen (mean age [± SD] 59.9 ± 14.3 years; range 17–76; 4 females) subjects with post-stroke hemiparesis for a mean duration (± SD) of 24.4 ± 40.5 months (range 0–166) participated in the study.

**Table 1:** Baseline characteristics and clinical data of subjects (n = 17)

Subject	Age	Sex	Time since stroke (months)	Affected side (side tested)	Diagnosis (Lesion)	MAS (average of two evaluators; out of 0–5)		BRS		Maximum angle of wrist dorsiflexion (passive)
						Wrist flexor	Finger flexor	Arm	Hand	
1	71	F	1	R	H (lt. putamen)	0	0	V	V	61
2	74	M	3	R	I (lt. putamen)	0	0.5	V	V	56
3	76	F	1	R	I (pons)	0.5	0	V	V	56
4	68	M	0	L	H (rt. subcortical)	0.5	0.5	V	V	49
5	59	M	40	R	I (lt. striatocapsular)	1	1	III–IV	IV	70
6	49	M	2	L	H (rt. thalamus)	1	0.5	V	V	57
7	57	M	36	R	I (lt. corona radiata)	1	0.5	V	V	60
8	72	M	4	R	I (lt. corona radiata)	1	1	V	V	51
9	17	M	22	R	H (lt. putamen)	1	2	III	III–IV	45
10	76	M	166	L	I (rt. MCA)	1.5	0.5	IV	V	57
11	59	F	2	L	H (rt.thalamus)	1.5	0.5	IV	V	57
12	66	M	5	L	H (rt. putamen)	2	2.5	III	III-IV	42
13	48	F	6	L	I (rt. MCA)	2	2	III	II	64
14	53	M	26	R	I (pons)	2	1.5	III–IV	III–IV	61
15	58	M	48	R	H (lt. putamen)	2.5	1	III	III	51
16	57	M	4	L	I (rt. MCA)	2.5	2	III	III	51
17	59	M	49	L	I (rt. lenticulostriate)	3	1.5	III	III	55

Brunnstrom Recovery Stage (BRS) classifies the motor recovery process for patients with stoke into six stages. This classification was established from clinical observations of a large number of hemiplegic patients and is based on the degree of synergy, voluntary movement and spasticity. In the BRS, staging is performed for each of the upper limbs, fingers, and lower limbs according to the criteria as follows: Stage I = Flaccidity is present and no movements of the limbs can be initiated; Stage II = The basic limb synergies or some of their components may appear as associated reactions or minimal voluntary movement responses may be present. Spasticity begins to develop; Stage III = The patient gains voluntary control of the movement synergies, although full range of all synergy components does not necessarily develop. Spasticity is severe; Stage IV = Some movement combinations that do not follow the synergies are mastered and spasticity begins to decline; Stage V = More difficult movement combinations are possible as the basic limb synergies lose their dominance over motor acts; Stage VI = Spasticity disappears and individual joint movements become possible

*M* male; *F* female; *I* infarction; *H* hemorrhage; *rt* right; *lt* left; *MAS* Modified Ashworth Scale; *BRS* Brunnstrom Recovery Stage

### Reliability

The single measure ICC (1,1) showed fair to good reliability in maximum RF, damping force, and damping impulses. However, ratio values such as damping force ratio and damping impulse ratio showed partly poor reliability. On the other hand, the average measure ICC (1, 10) was more than 0.75 for almost all parameters (Table 2).

**Table 2:** Intra rater reliability of various parameters for 10 repetitive meaurments

Parameters	ICC (1,1)(95%CI)			ICC(1,10)(95%CI)		
	30 deg/s	60 deg/s	90 deg/s	30 deg/s	60 deg/s	90 deg/s
Maximum RF	0.995 (0.990–0.998)	0.995 (0.990–0.998)	0.993 (0.987–0.997)	0.9995 (0.9990–0.9998)	0.999 (0.9990–0.9998)	0.9993 (0.9987–0.9997)
Damping force	0.797 (0.670–0.904)	0.895 (0.816–0.953)	0.848 (0.744–0.931)	0.975 (0.953–0.990)	0.988 (0.978–0.995)	0.982 (0.967–0.993)
(Total) damping impulse	0.974 (0.952–0.989)	0.972 (0.948–0.988)	0.970 (0.945–0.987)	0.997 (0.995–0.999)	0.997 (0.995–0.999)	0.997 (0.994–0.999)
(Pure) damping impulse	0.685 (0.525–0.841)	0.823 (0.706–0.917)	0.813 (0.692–0.912)	0.956 (0.917–0.981)	0.979 (0.960–0.991)	0.977 (0.957–0.991)
Damping force ratio	0.557 (0.382–0.757)	0.621 (0.451–0.801)	0.754 (0.612–0.881)	0.926 (0.861–0.969)	0.941 (0.891–0.976)	0.968 (0.940–0.987)
Damping impulse ratio	0.480 (0.306–0.699)	0.391 (0.227–0.624)	0.629 (0.459–0.806)	0.902 (0.815–0.959)	0.865 (0.746–0.943)	0.944 (0.895–0.976)

Cohen’s kappa coefficient for the MAS in wrist flexors (κ = 0.44, 95% CI 0.30–0.57, p = 0.002), finger flexors (κ = 0.14, 95% CI 0.0003–0.28, p = 0.19), and the average of wrist and finger flexors combined (κ = 0.11, 95% CI 0.007–0.21, P = 0.13) showed poor inter-rater reliability.

### Timing of peak resistance

In this study, there was a 2-s pause from reaching the maximum extension angle to the start of subsequent flexion. Since the average (± SD) duration from the peak resistance to the start of the next flexion was 1.84 ± 0.11 s at an angular velocity of 30 deg/s, 1.77 ± 0.17 s at 60 deg/s, and 1.79 ± 0.15 s at 90 deg/s, the peak resistance force was observed after the extension angle reached the target (maximum) angle.

### Velocity-dependence of various parameters

**Fig. 3:**
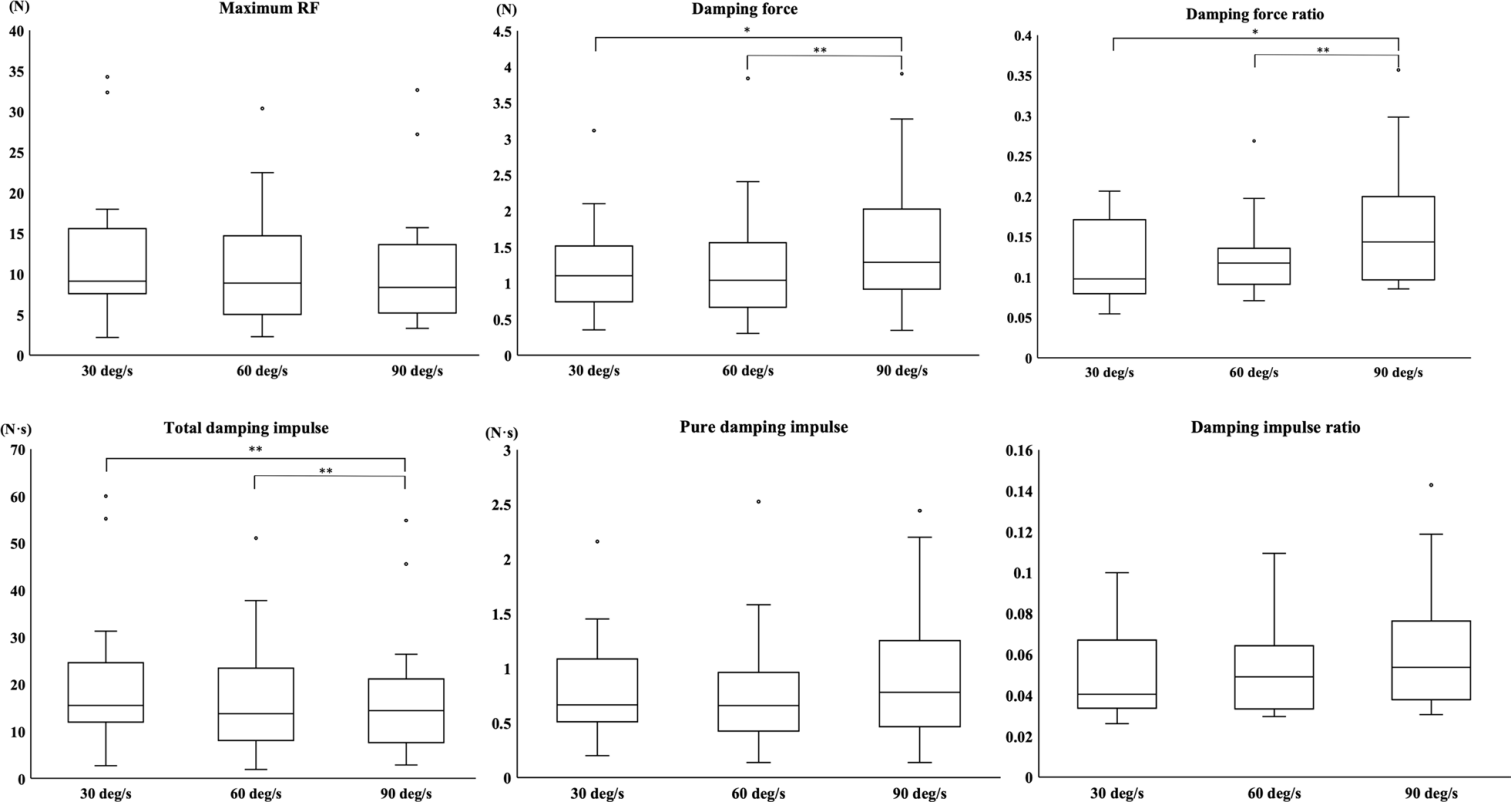
Comparisons of various parameters among the 3 velocity conditions. The lower boundary of each box indicates the 25th percentile, the line within the box marks the median, and the upper boundary indicates the 75th percentile. Whiskers above and below each box indicate the maximum and minimum values, respectively. The open circles denote outliers. The Shapiro–Wilk test showed that not all data were normally distributed for each parameter. The nonparametric Friedman test followed by the post-hoc Wilcoxon signed-rank test with Holm’s correction was applied. The asterisks indicate significant differences (*P < 0.05, **P < 0.01) between conditions

The median of the maximum RF at the 3 different velocities was 9.11 N at 30 deg/s, 8.89 N at 60 deg/s, and 8.33 N at 90 deg/s. Although there was no significant difference between the maximum RF at different velocities, these values tended to decrease as the velocities increased (Friedman χ^2^ = 4.6, df = 2, p-value = 0.10) (Fig. 3). The damping force showed a significant increase (χ^2^ = 10.7, df = 2, p-value < 0.01) as the velocities increased, except between 30 and 60 deg/s, and there was no significant difference in the pure damping impulse (χ^2^ = 2.2, df = 2, p-value = 0.33) (Fig. 3). The total damping impulse showed a significant decrease (χ^2^ = 12.8, df = 2, p-value < 0.01). The damping force ratio (B/A) showed a significant velocity-dependent increase (χ^2^ = 14.6, df = 2, p-value < 0.001) except between 30 and 60 deg/s, and the damping impulse ratio (D/C) tended to increase with velocity (χ^2^ = 5.6, df = 2, p-value = 0.059), as shown in Fig. 3.

When we compared the first and 10th values for each parameter at each angular velocity (i.e., the second and 11th values of 11th consecutive measurements), there was a significant decrease for all angular velocities only in the Maximum RF (Table 3).

**Table 3:** Comparison of the 1st and 10th (2nd and 11th of 11 repetition) measured values at each velocity in various parameters (average ± SD)

Angular velocity	Maximum RF (N)		P value	Damping force (N)		P value	Damping force ratio		P value
	**1st**	**10th**		**1st**	**10th**		**1st**	**10th**	
30 deg/s	13.0 ± 9.4	12.1 ± 8.8	< 0.05 (W)	1.33 ± 0.76	1.10 ± 0.69	p < 0.05 (W)	0.12 ± 0.044	0.11 ± 0.064	p = 0.49 (W)
60 deg/s	11.3 ± 7.8	10.6 ± 7.1	< 0.01 (P)	1.41 ± 1.04	1.16 ± 0.82	p = 0.13 (W)	0.14 ± 0.070	0.13 ± 0.086	p = 0.58 (W)
90 deg/s	11.5 ± 8.0	10.9 ± 7.9	< 0.01 (W)	1.64 ± 1.04	1.60 ± 1.07	p = 0.75 (P)	0.16 ± 0.085	0.16 ± 0.079	p = 0.93 (P)

Angular velocity	Pure dumping impulse (N·s)		P value	Total dumping impulse (N·s)		P value	Damping impulse ratio		P-value
	1st	10th		1st	10th		1st	10th	
30 deg/s	0.93 ± 0.59	0.73 ± 0.50	p < 0.05 (W)	22.16 ± 17.45	20.45 ± 15.71	p = 0.098 (W)	0.050 ± 0.018	0.044 ± 0.026	p = 0.063 (W)
60 deg/s	0.92 ± 0.76	0.73 ± 0.58	p = 0.12 (W)	18.56 ± 13.40	17.74 ± 12.46	p = 0.14 (P)	0.054 ± 0.033	0.054 ± 0.045	p = 0.40 (W)
90 deg/s	0.93 ± 0.63	0.88 ± 0.70	p = 0.49 (W)	18.12 ± 14.48	16.97 ± 13.68	p = 0.051 (W)	0.060 ± 0.029	0.057 ± 0.025	p = 0.41 (P)

Shapiro–Wilk normality test was applied to check the distribution of data

For non parametric data, Wilcoxon signed rank test (W) was conducted, otherwise paired t test (P) was conducted

### Correlation between each parameter and the average MAS

We investigated the correlations of the maximum RF, the damping force, the pure damping impulse, the total damping impulse, the damping force ratio, and the damping impulse ratio with the clinically scored muscle spasticity according to the average MAS (the average scores of the MAS obtained by two therapists only for the wrist flexors and for the wrist and finger flexors combined [wrist and finger flexors combined values were divided by 2 to calculate the average value per joint]). No significant correlations were found between the maximum RF, the damping force, or the damping impulse and the average MAS. On the other hand, there were significant (p < 0.05) correlations between the damping force ratio and the damping impulse ratio and both the average MAS for wrist flexors (*ρ* = 0.53–0.56) at velocity of 90 deg/s, and the average MAS for wrist and finger flexors (*ρ* = 0.50–0.68) at velocities of 60 deg/s and 90 deg/s, respectively. However, neither the damping force ratio nor the damping impulse ratio was significantly correlated with the average MAS at 30 deg/s (Table 4). Since the average MAS showed higher correlations with both ratio parameters at 90 deg/s compared to at 60 deg/s, the correlation coefficient *ρ* tended to increase in a velocity-dependent manner. Furthermore, if we compare the correlations between these ratio parameters and the average MAS scores for wrist flexors to those for both wrist and finger flexors combined, the latter tended to be more relevant (Table 4).

**Table 4:** Correlation (ρ value) between the average MAS and various parameters for each achieved velocity^a^

	Maximum RF			Damping force			Total damping impulse			Pure damping impulse			Damping force ratio			Damping impulse ratio		
	Angular velocity (deg/s)			Angular velocity (deg/s)			Angular velocity (deg/s)			Angular velocity (deg/s)			Angular velocity (deg/s)			Angular velocity (deg/s)		
	30	60	90	30	60	90	30	60	90	30	60	90	30	60	90	30	60	**90**
Average MAS																		
Wrist	0.03	0.01	0.01	0.3	0.28	0.38	0.03	− 0	− 0.04	0.24	0.3	0.36	0.23	0.43	0.53*	0.25	0.47	0.56*
Wrist + finger	− 0.13	− 0.2	− 0.17	0.16	0.09	0.32	− 0.13	− 0.18	− 0.18	0.13	0.16	0.27	0.3	0.50*	0.65**	0.36	0.57*	0.68**

^a^*P < 0.05; **P < 0.01 Spearman rank correlation test was applied for all data analysis

### Differences depending on the severity of spasticity

**Fig. 4:**
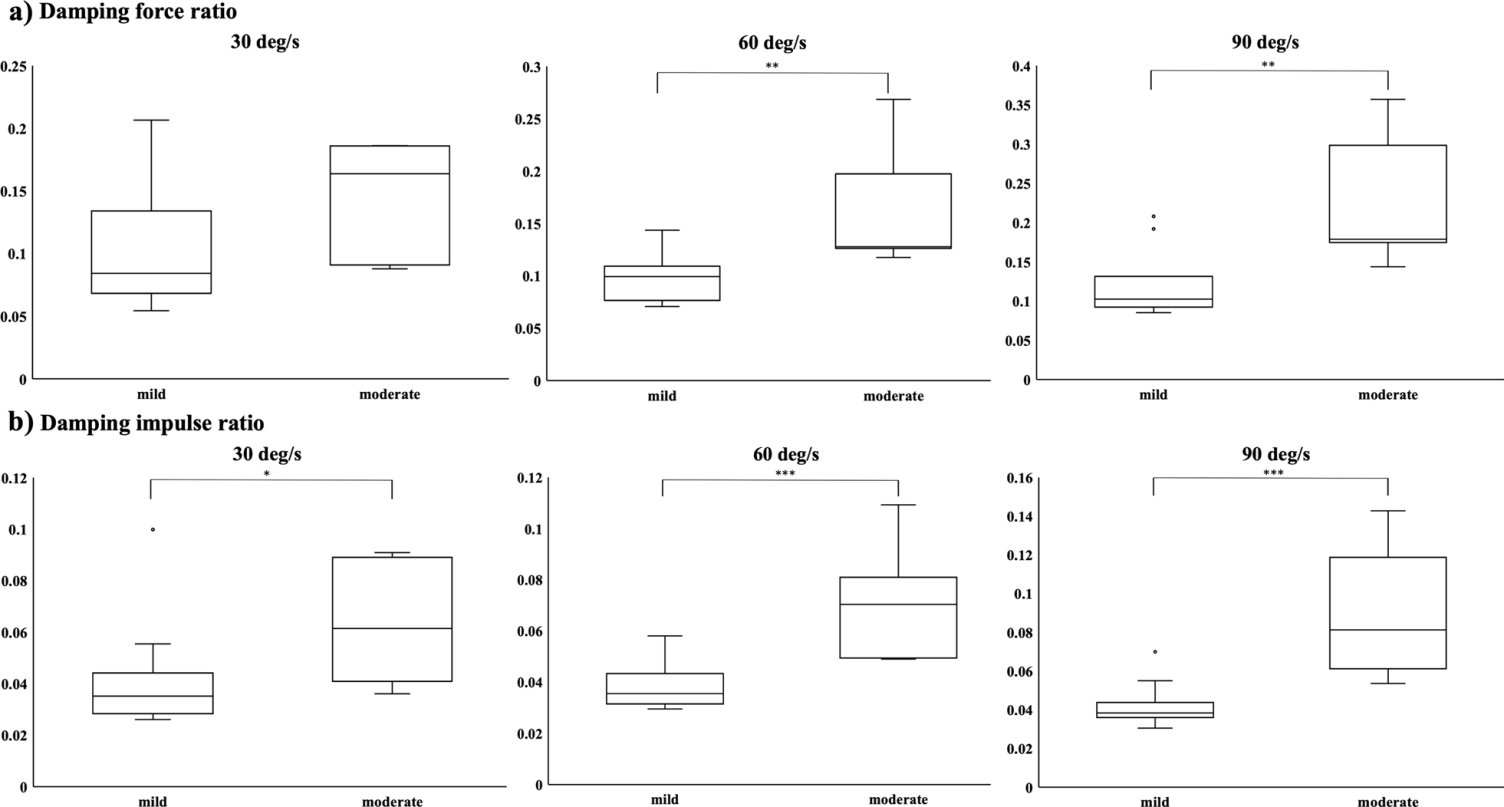
Comparisons between mild and moderate spasticity. In each of the 3 velocity conditions for the damping force ratio (**a**) and the damping impulse ratio (**b**). The asterisks indicate significant differences (*P < 0.05, **P < 0.01, ***P < 0.001) between groups. The Shapiro–Wilk and Levene statistical tests were used to examine the normality and equality of variance. According to the results of these tests, a two-sample t test (TT) or Wilcoxon-Mann–Whitney test (WMW) was applied for between-group analysis. The differences at 90 deg/s for both ratios and at 30 deg/s for the damping impulse ratio were analyzed by WMW; otherwise, TT was used

The differences in each parameter were investigated when the patients were divided into two groups based on the average MAS scores (average value per joint) in the wrist and finger flexors assessed by the two evaluators, where a score of 1or less (out of 0–5) was considered mild spasticity and more than 1 was considered moderate spasticity. While no significant difference in maximum RF was seen between the two groups at each velocity, both the damping force ratio and the damping impulse ratio were significantly higher in the moderate spasticity group than in the mild group at 60 deg/s (P = 0.004, TT) and 90 deg/s (P = 0.007, WMW) for the damping force ratio, and at 30 deg/s (P = 0.03, WMW), 60 deg/s (P = 0.0009, TT), and 90 deg/s (P = 0.0007, WMW) for the damping impulse ratio (Fig. 4).

## Discussion

In the present study, we applied mechanical passive stretch with a custom-made motor-controlled device to the hemiparetic wrist in 17 participants with spasticity following stoke. The isokinetic passive extensive displacement of wrist flexor muscles with the device induced resistant reflex forces and subsequent depression of these forces. The indices obtained from maximum RF and the subsequent force reduction after maximum extension (damping force ratio and damping impulse ratio) increased in a velocity-dependent manner and showed significant velocity-dependent correlations with average MAS scores. When the MAS evaluation for the finger flexors was added to the MAS for the wrist flexors, the correlations became stronger. Furthermore, when the patients were divided into a mild group (average MAS 1or less) and a moderate group (average MAS more than 1), both the damping force ratio and the damping impulse ratio showed significantly higher values in the moderate group at angular velocities of 60 deg/s and 90 deg/s. Although MAS shows poor reliability at the lower end of the scale [14] and all of the patients in this study had average MAS scores of 3 (out of 0–5) or less in both fingers and wrist flexors, significant differences were detected between the mild and moderate groups at 60 deg/s and 90 deg/s. Measurements of the resistance to wrist extension with the current custom device may have detected changes similar to those felt in evaluating stiffness with MAS in a daily clinical setting, especially in patients with comparatively mild spasticity.

Previous studies measured joint stiffness including spasticity by considering a passive resistance force or torque in the wrist [34–37, 47], finger [38], elbow [30, 48], and ankle joints [25–29]. However, no studies have shown the correlation between the attenuated resistance force after the resistance peak and the MAS in hemiparetic patients with mild to moderate spasticity. This is the first report that focuses on the damping force after the peak resistance force induced by passive movement with isokinetic and motor-controlled velocity.

We often encounter a rapid rise of resistance in the latter half of the range of motion and subsequent weakening of resistance in patients with mild spasticity, when spastic muscle tone is measured manually in the clinic. In addition, the so-called clasp-knife phenomenon or the definition of the MAS (1 and 1 +), which refers to the attenuation of resistance after an increase in resistance, has been known for a long time and is considered to reflect the subsidence of electromyogram and resistance force [23, 40].

Although the force output by our device is probably different from that applied when spasticity is evaluated in the daily clinical setting, the measured changes in damping with isokinetic passive movements using the current device might reflect the mechanical changes that constitute the clasp-knife phenomenon and catch and release which we feel when evaluating a patient’s spasticity with MAS.

### Normalization procedure

We calculated the ratio of the damping force to the maximum RF and that of the pure damping impulse to the total damping impulse, which may contribute to normalize the fluctuation of resistance due to the sliding mechanism, the effect of the setting position, and the difference in mechanical responsiveness to passive movement between individuals. Several studies have investigated the correlation between resistance to passive movement in various joints and the Ashworth Scale and MAS using a similar device [25–29, 34–36]. In addition, several studies have applied some ratio indices for normalized values [26, 27, 35], as we did in the current study. The correlation with MAS was clarified [27] and the difference between spastic and non-spastic patients in low-velocity passive movement was verified [26] only when the analysis was performed using normalized values.

In this study, normalization of the damping force and impulse through the use of a ratio might contribute to detect the difference at relatively low velocities, and to identify significant correlations with the average MAS.

On the other hand, there is an interesting study in which normalization was performed by subtracting the measured baseline torque at a very low speed which induced no spastic response based on the torque measured at a higher speed [38]. This method is considered to be a useful normalization that can remove the mechanical changes other than reflex torque that occur with passive extension. We expect that this normalization could cancel out the extra mechanical changes associated with the sliding mechanism in our device, and, in combination with the ratio normalization used in our study, it may result in a more rigorous extraction of the spasticity component.

In studies using mechanical resistance, it might sometimes be important to apply some normalization procedures when comparing resistance to clarify the differences between individual subjects.

### Velocity-dependent resistance and correlation with the MAS

Spasticity has been defined as a velocity-dependent increase in resistance during passive stretch. Previous studies have reported that the maximum resistance against passive movements increased in a velocity-dependent manner [26, 27, 36, 49] and was correlated with the MAS [36]. However, in the current study, the maximum RF tended to decrease with velocity, while the normalized damping part tended to increase and showed significant correlations with the MAS in a velocity-dependent manner. One of the reasons why the resistance decreased in a speed-dependent manner in this study could be that measurements were performed in the order 30 deg/s, 60 deg/s, and 90 deg/s with a short interval. Previous studies have indicated that repetitive passive movements reduced reflex torque [50] in elbow flexors and post-activation depression in the soleus muscle [51]. In the wrist and finger flexors, the long latency component of the stretch reflex decreases at faster repetition rates [52]. As in the current study, it has been reported that resistance decreased in a velocity-dependent manner in patients with mild spasticity after stroke and was not correlated with MAS [40]. However, in the current study, we detected a velocity-dependent increasing tendency by focusing on the damping part, which has not been a focus of previous studies, and found significant correlations with MAS.

In previous studies, electromyographical and resistance changes in spastic muscles were investigated at various passive movement velocities from 5 to 500 deg/s in the wrist joint [34, 36, 37, 47, 53–55]. Among these studies, a few found a significant correlation between the velocity-dependent resistance component and MAS at low-velocity joint movements of 50–70 deg/s [36, 53], and our study also supports these findings. On the other hand, since the correlations and differences observed in the present study are more pronounced in a speed-dependent manner, further measurements at higher speeds may reveal larger damping changes and lead to more valid measurement results.

In the current study, the correlation between the damping part and the MAS became stronger when the MAS in finger flexors was added to the MAS in wrist flexors. Since the extrinsic finger muscles affect the passive component of wrist joint stiffness [55–57], it was considered that the stiffness of the extrinsic flexors of the fingers fixed to the device in the extended position may further affect the resistance to wrist extension. These changes in the correlations may indicate that the measured changes in force in the damping part quantitatively detect the spastic changes in wrist and finger flexors simultaneously. Typically, wrist spasticity is measured manually without all fingers extended. Since this device measures with the fingers extended, if spasticity of the extrinsic muscles of the fingers, such as the flexor digitorum superficialis (FDS) and flexor digitorum profundus (FDP), is strong, it is likely to reflect more spasticity in the finger flexors than in the wrist flexors, so care must be taken when interpreting measured values. If the equipment is modified to measure passive resistance force in both the extended and flexed finger positions, it may be possible to calculate spasticity of the extrinsic finger flexor muscles in addition to spasticity of the wrist flexors.

Kamper et al. [38] have shown in their finger spasticity measurement studies that the reduction in torque immediately after extrinsic finger flexors muscle extension caused by concurrent involuntary activity of the extensor digitorum communis (EDC). This finding has major implications for the “damping” that has been a focus of our research. Since the fingers are fixed to the hand plate in the extended position during measurement with our device, it is thought that the FDS and FDP are extended at the same time as passive extension of the wrist flexor muscles. Therefore, it is possible that the EDC muscle activity may have been evoked and influenced the damping force measured in this study. If EMG measurements of both flexor and extensor muscles can be performed simultaneously with our device, and if the aforementioned measurements during finger flexion and extension can be compared, this should provide a better understanding of the changes in damping forces associated with wrist flexor and finger flexor extension.

### Force components that make up the damping force

The damping part that we focused on might be mainly associated with a neural component that includes spasticity. According to several studies, the resistance generated by passive wrist dorsiflexion consists of inertia, elasticity, viscosity, and neural components caused by muscle stretch reflexes [36, 48]. In post-stroke patients, Lindberg et al. [36] showed that there are individual differences in the magnitude of passive resistance and in the composition of each component. However, for these components, the viscosity and neural components change in a velocity-dependent manner, and the elastic component continues with constant resistance and increases at the end of the range of extension. Additionally, the proportion of neural components in the total resistance is relatively large [36]. On the other hand, inertia appears and disappears for a short time at the start of the increase in resistance and just before reaching the maximum range of extension [27, 36]. Based on these reports, the resistance damping part that we focused on in the current study could have arisen from both the viscosity and the neural components and the damping part might be mainly associated with the neural component including spasticity, since that part depended on velocity and appeared after the wrist joint reached the maximum range of extension.

### Limitations

In several previous studies that measured torque and force for the assessment of joint stiffness or spasticity, the measurements of passive resistance were conducted only after sufficient time intervals. In the current study, continuous sessions with pauses of only several seconds were performed with a short rest of 1 to 2 min in the ascending order of velocity (30 deg/s, 60 deg/s, and 90 deg/s) in all subjects. The resistance forces were considered to be affected by attenuation due to repetition. Decrease of maximum RF by repetitive measurement at each velocity (Table 3) and the significant velocity-dependent decrease in total damping impulse (Fig. 3) may indicate that the overall resistance change decreased with repetition. The measurement intervals could have been longer [27, 36] and the angular velocities could have been measured randomly [26] or in a different order [36].

In the intra-rater reliability test, the reliability of the single measurement was partially poor, especially for the ratio index. To obtain more reliable data with less patient burden, it is necessary to examine measurement methods to obtain reliable results with fewer repetitions.

The ingenuity of the sliding mechanism could contribute to cancel out excessive tension on the wrist joint, while the resistance force fluctuated due to movement of the positional relationship of the hand with respect to the pressure-measurement part of the tension/compression load cell. Furthermore, when the hand and forearm were fixed to the device, misalignment of the wrist joint axis could occur, and therefore it would be necessary to address inter-rater reliability and the measurements in healthy controls.

Some studies have questioned the reliability and validity of the MAS used in this study [58, 59]. In an attempt to improve the reliability of MAS measurement data, measurements were taken by two therapists. However, inter-rater reliability for the two measurers in this study was also poor for both wrist and finger flexors.

Ansari et al. modified the MAS by deleting grade 1 + and redefining grades 1 and 2, to give the Modified Modified Ashworth Scale (MMAS), which has an ordinal relationship [58, 60, 61]. In the MMAS, the modified portion is defined as follows: 1 = slight increase in muscle tone, manifested by a catch and release or by minimal resistance at the end of the ROM when the affected part is moved in flexion or extension; 2 = marked increase in muscle tone, manifested by a catch in the middle range and resistance throughout the remainder of the ROM, but the affected part is easily moved. Given the reported good reliability [62, 63] and validity [64] of the MMAS for wrist flexors, we expect that this scale can be used to more accurately validate the measurements of this device.

In the analysis in this study, it is somewhat problematic that MAS, which is a nominal measure, was treated as an ordinal measure. Also, since the sample size was small, it would have been better to examine a larger number of subjects in stratified or categorized analyses.

## Conclusion

Our custom-made isokinetic device could be used to perform a quantitative evaluation of spasticity by focusing on the damping part following increased resistance. In addition, the damping part might be equivalent to the decrease in resistance we perceive when assessing wrist spasticity manually using MAS.

Especially for patients with mild to moderate spasticity, focusing on the damping part measured with this device may make it possible to objectively, stably and safely detect differences in stiffness including spasticity with isokinetic passive movements at a low angular velocity. In the field of rehabilitation, to improve the ability to control the wrist and fingers of patients with spastic hemiparesis after stroke with higher dexterity, it is very important to accurately evaluate these stiffnesses and control them by therapeutic intervention. This device, which could simultaneously reflect spastic changes in both the wrist and finger flexors, may be effectively used in the diagnosis and treatment of various neurological diseases.

In order for this device to be used as part of a routine examination in clinics, it is not enough to demonstrate its effectiveness and safety. Both therapists and patients must be able to set up and take measurements with the device quickly and easily, and the cost must be kept down [65]. To overcome these challenges, it will be necessary to consider modification of measurement methods and the device, and there are still many issues to be addressed in the future.

## Data Availability

The datasets are available from the corresponding author on reasonable request.

## Abbreviations

MAS: Modified Ashworth Scale
ROM: Range of motion
maximum RF: Maximum resistance force
ICC: Intraclass correlation coefficient
ANOVA: Analysis of variance
TT: Two-sample t test
WMW: Wilcoxon-Mann–Whitney test
